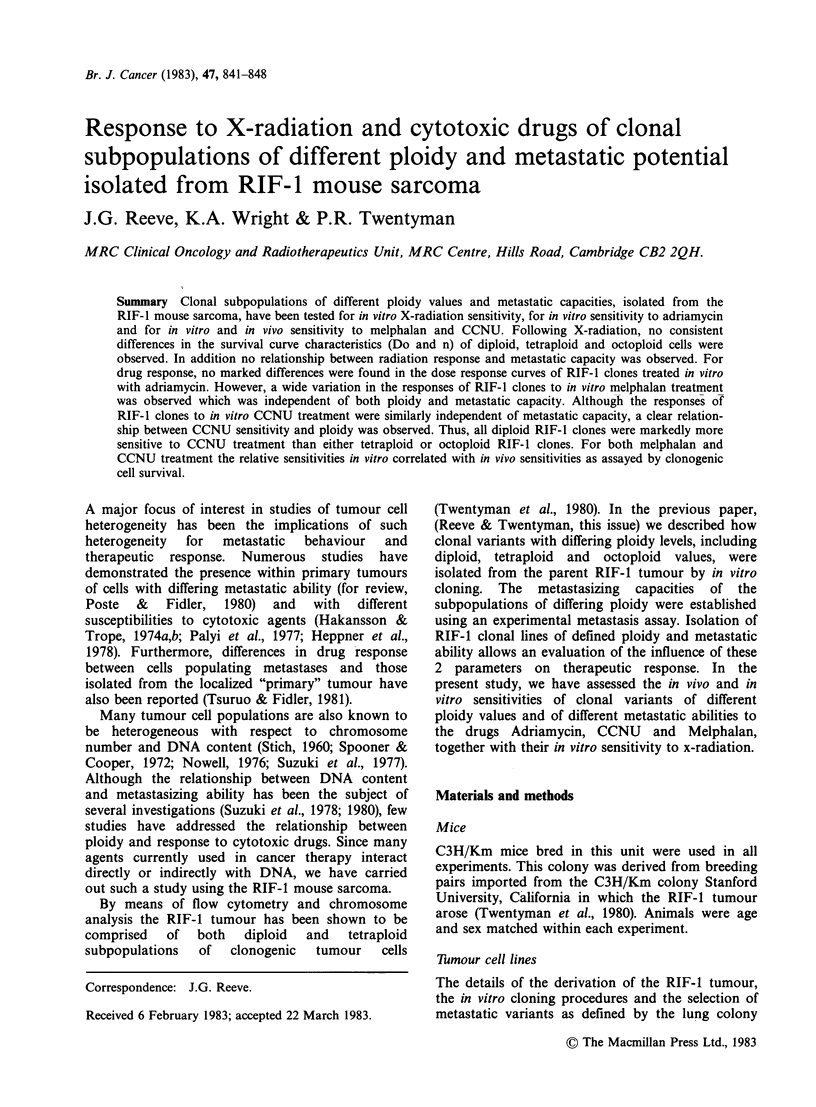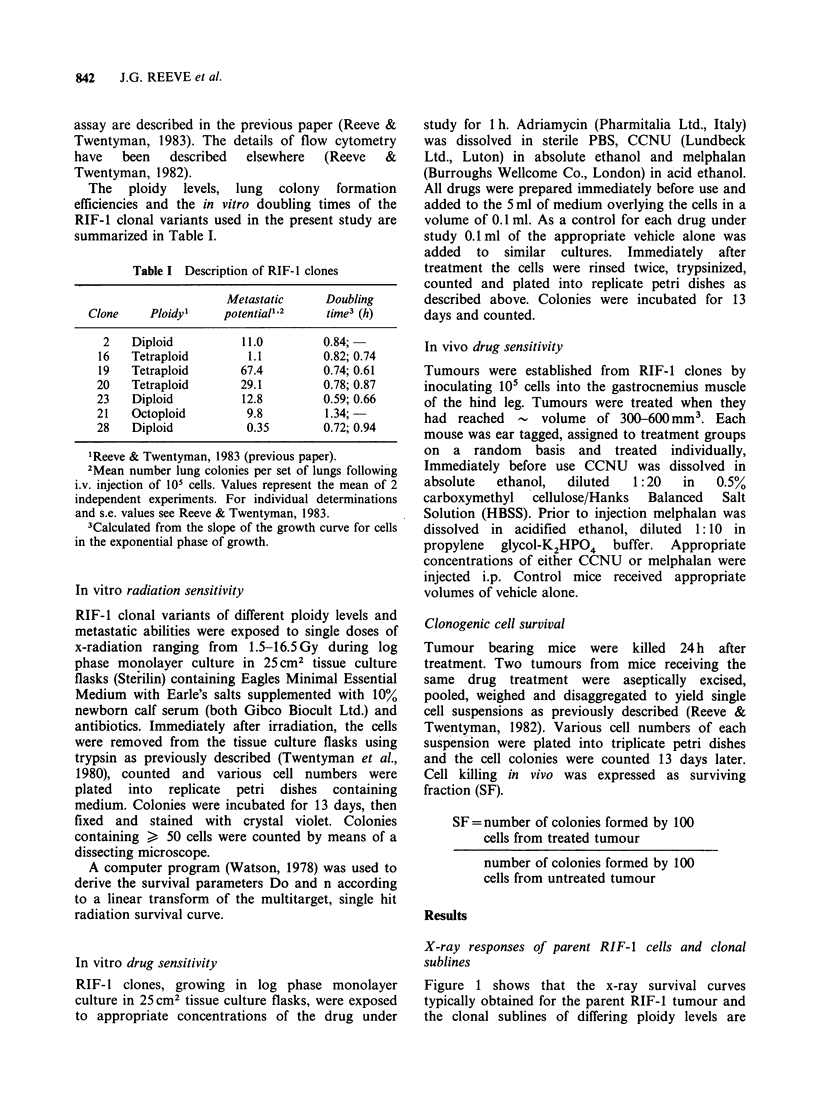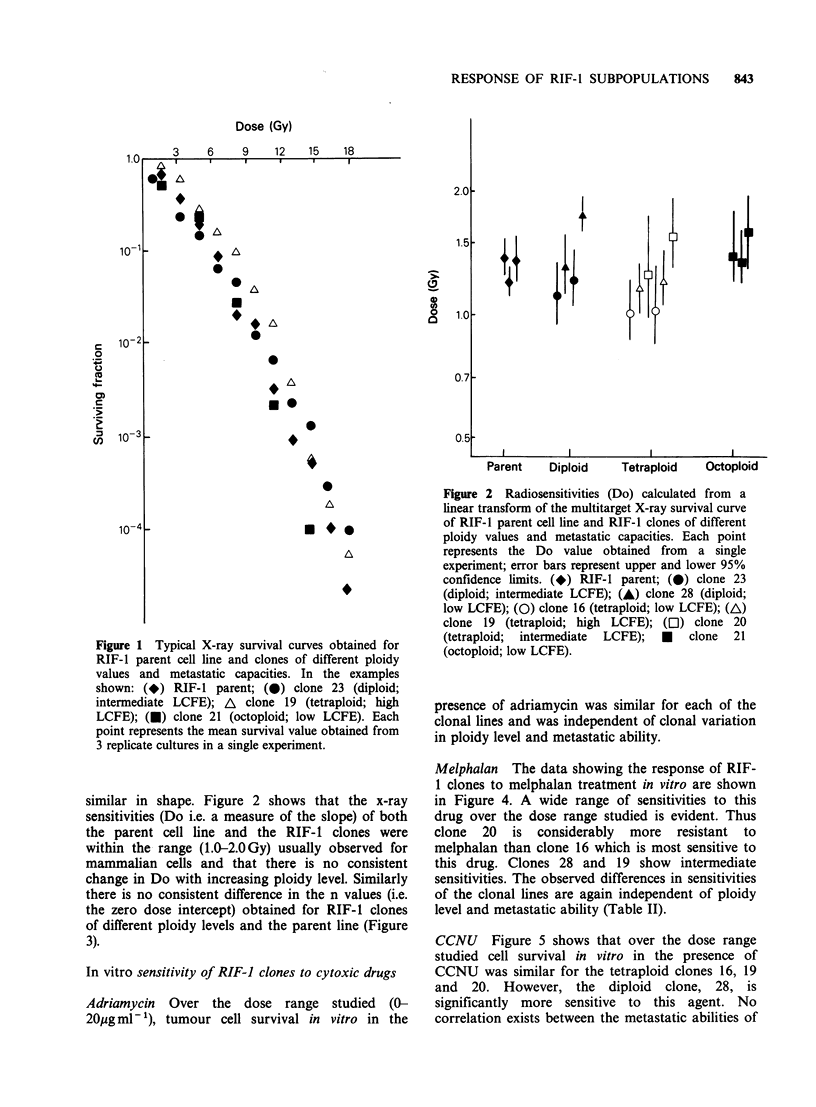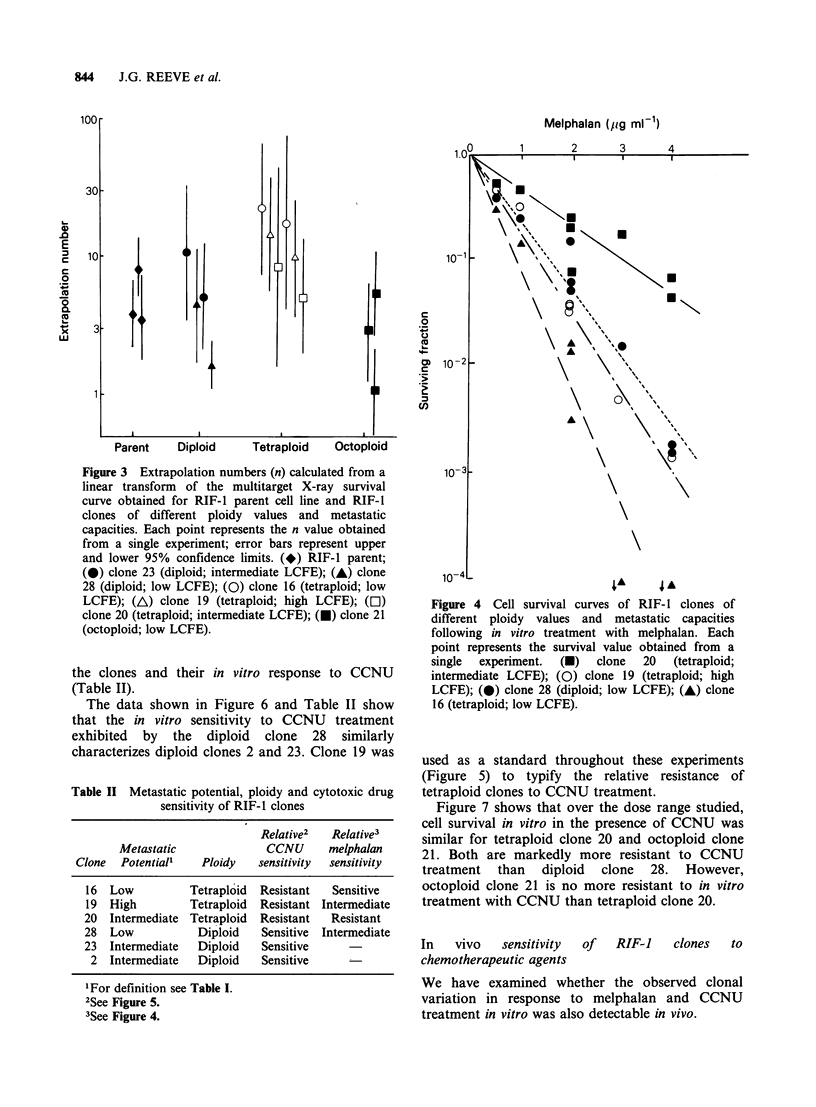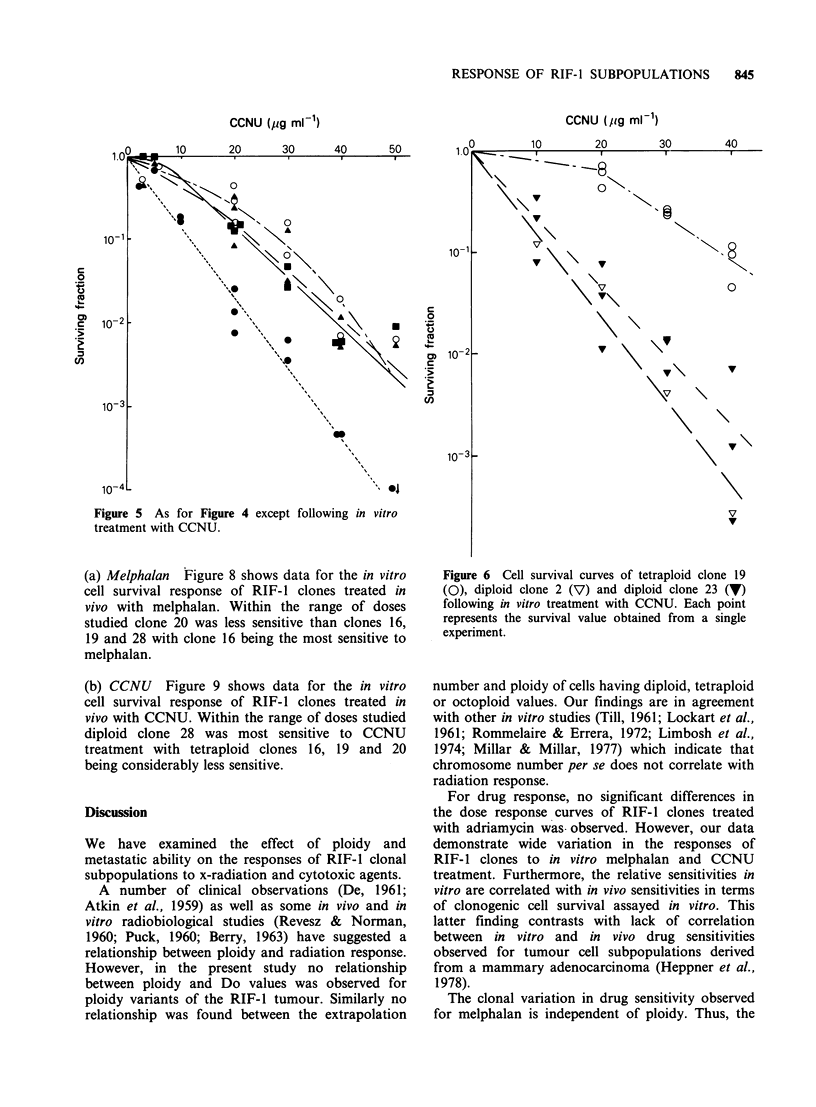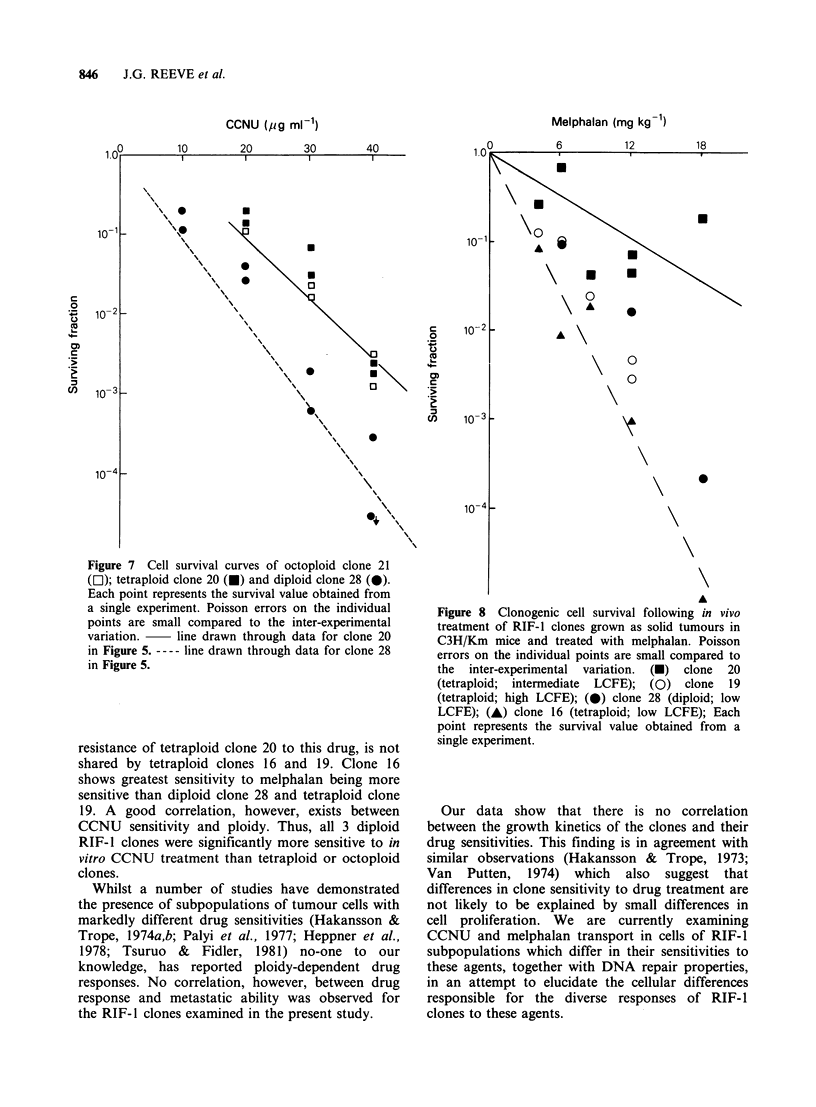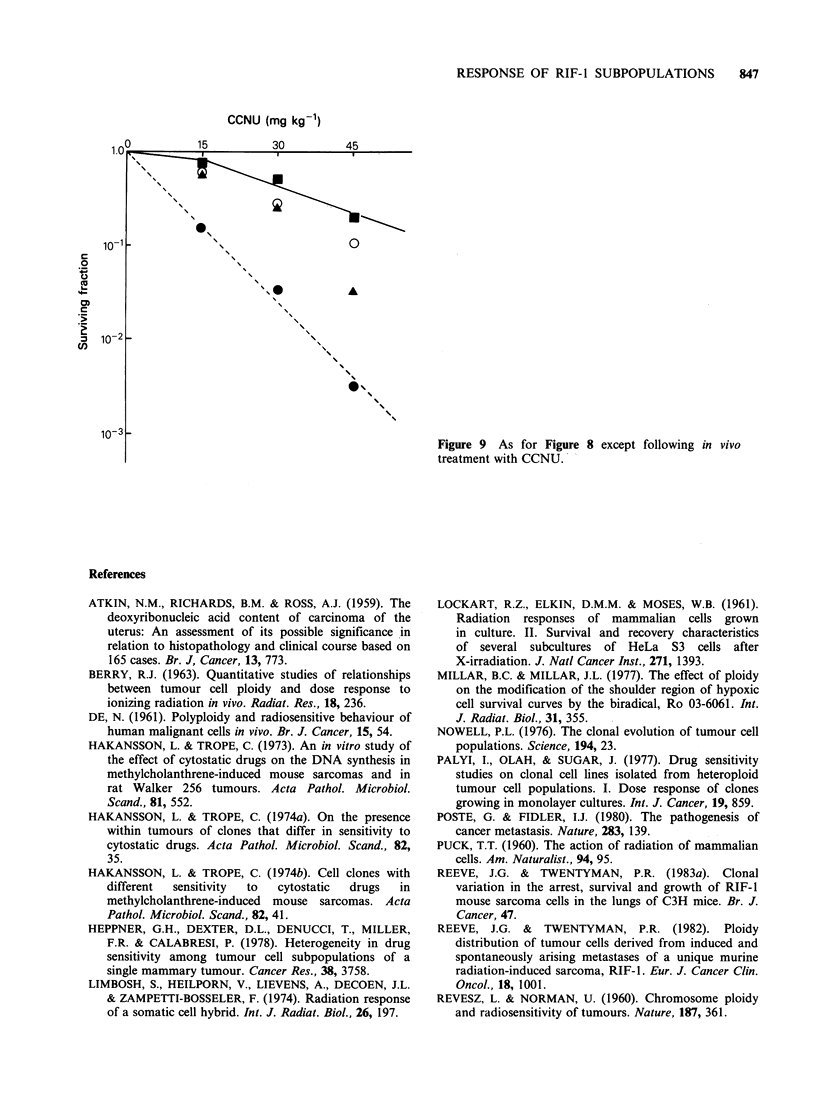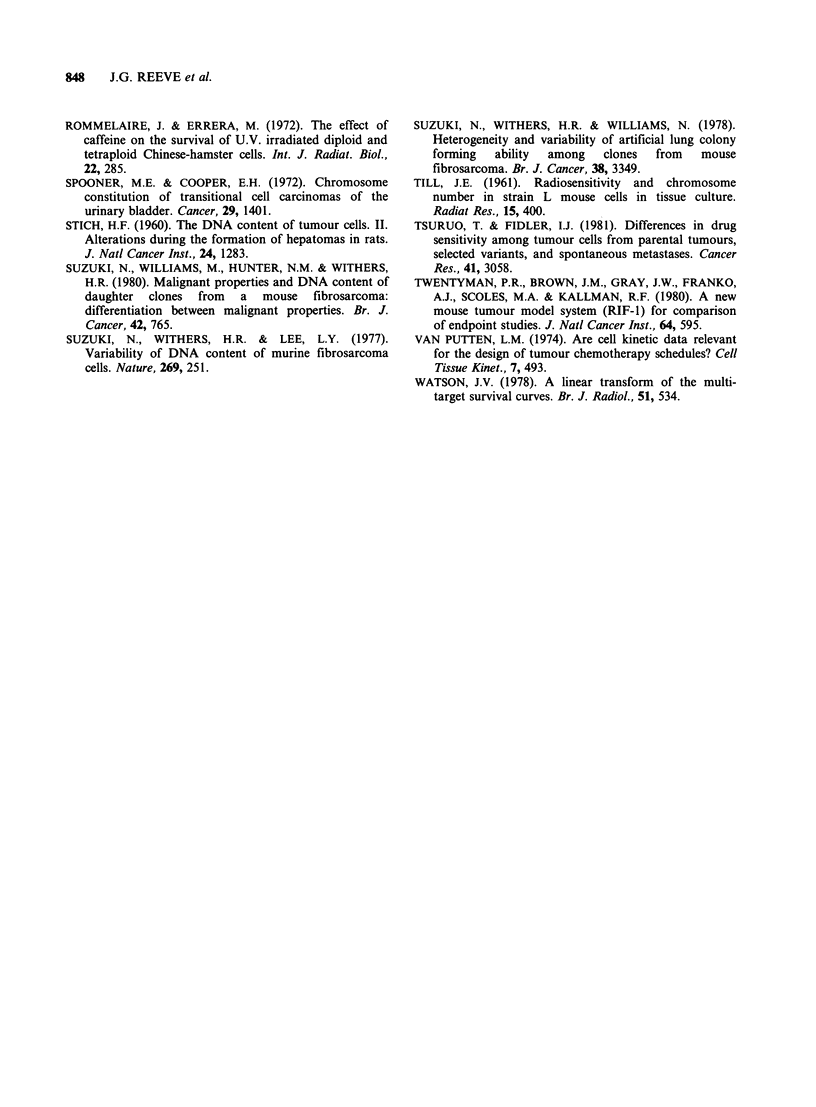# Response to X-radiation and cytotoxic drugs of clonal subpopulations of different ploidy and metastatic potential isolated from RIF-1 mouse sarcoma.

**DOI:** 10.1038/bjc.1983.139

**Published:** 1983-06

**Authors:** J. G. Reeve, K. A. Wright, P. R. Twentyman

## Abstract

Clonal subpopulations of different ploidy values and metastatic capacities, isolated from the RIF-1 mouse sarcoma, have been tested for in vitro X-radiation sensitivity, for in vitro sensitivity to adriamycin and for in vitro and in vivo sensitivity to melphalan and CCNU. Following X-radiation, no consistent differences in the survival curve characteristics (Do and n) of diploid, tetraploid and octoploid cells were observed. In addition no relationship between radiation response and metastatic capacity was observed. For drug response, no marked differences were found in the dose response curves of RIF-1 clones treated in vitro with adriamycin. However, a wide variation in the responses of RIF-1 clones to in vitro melphalan treatment was observed which was independent of both ploidy and metastatic capacity. Although the responses of RIF-1 clones to in vitro CCNU treatment were similarly independent of metastatic capacity, a clear relationship between CCNU sensitivity and ploidy was observed. Thus, all diploid RIF-1 clones were markedly more sensitive to CCNU treatment than either tetraploid or octoploid RIF-1 clones. For both melphalan and CCNU treatment the relative sensitivities in vitro correlated with in vivo sensitivities as assayed by clonogenic cell survival.


*Br. J. Cancer* (1983), **47**, 841–848

### Response to X-radiation and cytotoxic drugs of clonal subpopulations of different ploidy and metastatic potential isolated from RIF-1 mouse sarcoma

J.G. Reeve, K.A. Wright & P.R. Twentyman

*MRC Clinical Oncology and Radiotherapeutics Unit, MRC Centre, Hills Road, Cambridge CB2 2QH.*

**Summary** Clonal subpopulations of different ploidy values and metastatic capacities, isolated from the RIF-1 mouse sarcoma, have been tested for *in vitro* X-radiation sensitivity, for *in vitro* sensitivity to adriamycin and for *in vitro* and *in vivo* sensitivity to melphalan and CCNU. Following X-radiation, no consistent differences in the survival curve characteristics (Do and n) of diploid, tetraploid and octoploid cells were observed. In addition no relationship between radiation response and metastatic capacity was observed. For drug response, no marked differences were found in the dose response curves of RIF-1 clones treated *in vitro* with adriamycin. However, a wide variation in the responses of RIF-1 clones to *in vitro* melphalan treatment was observed which was independent of both ploidy and metastatic capacity. Although the responses of RIF-1 clones to *in vitro* CCNU treatment were similarly independent of metastatic capacity, a clear relationship between CCNU sensitivity and ploidy was observed. Thus, all diploid RIF-1 clones were markedly more sensitive to CCNU treatment than either tetraploid or octoploid RIF-1 clones. For both melphalan and CCNU treatment the relative sensitivities *in vitro* correlated with *in vivo* sensitivities as assayed by clonogenic cell survival.

A major focus of interest in studies of tumour cell heterogeneity has been the implications of such heterogeneity for metastatic behaviour and therapeutic response. Numerous studies have demonstrated the presence within primary tumours of cells with differing metastatic ability (for review, Poste & Fidler, 1980) and with different susceptibilities to cytotoxic agents (Hakansson & Trope, 1974*a,b*; Palyi *et al.*, 1977; Heppner *et al.*, 1978). Furthermore, differences in drug response between cells populating metastases and those isolated from the localized "primary" tumour have also been reported (Tsuruo & Fidler, 1981).

Many tumour cell populations are also known to be heterogeneous with respect to chromosome number and DNA content (Stich, 1960; Spooner & Cooper, 1972; Nowell, 1976; Suzuki *et al.*, 1977). Although the relationship between DNA content and metastasizing ability has been the subject of several investigations (Suzuki *et al.*, 1978; 1980), few studies have addressed the relationship between ploidy and response to cytotoxic drugs. Since many agents currently used in cancer therapy interact directly or indirectly with DNA, we have carried out such a study using the RIF-1 mouse sarcoma.

By means of flow cytometry and chromosome analysis the RIF-1 tumour has been shown to be comprised of both diploid and tetraploid subpopulations of clonogenic tumour cells (Twentyman *et al.*, 1980). In the previous paper, (Reeve & Twentyman, this issue) we described how clonal variants with differing ploidy levels, including diploid, tetraploid and octoploid values, were isolated from the parent RIF-1 tumour by *in vitro* cloning. The metastasizing capacities of the subpopulations of differing ploidy were established using an experimental metastasis assay. Isolation of RIF-1 clonal lines of defined ploidy and metastatic ability allows an evaluation of the influence of these 2 parameters on therapeutic response. In the present study, we have assessed the *in vivo* and *in vitro* sensitivities of clonal variants of different ploidy values and of different metastatic abilities to the drugs Adriamycin, CCNU and Melphalan, together with their *in vitro* sensitivity to x-radiation.

Correspondence: J.G. Reeve.

Received 6 February 1983; accepted 22 March 1983.

### Materials and methods

### *Mice*

C3H/Km mice bred in this unit were used in all experiments. This colony was derived from breeding pairs imported from the C3H/Km colony Stanford University, California in which the RIF-1 tumour arose (Twentyman *et al.*, 1980). Animals were age and sex matched within each experiment.

### *Tumour cell lines*

The details of the derivation of the RIF-1 tumour, the *in vitro* cloning procedures and the selection of metastatic variants as defined by the lung colony

© The Macmillan Press Ltd., 1983

assay are described in the previous paper (Reeve & Twentyman, 1983). The details of flow cytometry have been described elsewhere (Reeve & Twentyman, 1982).

The ploidy levels, lung colony formation efficiencies and the *in vitro* doubling times of the RIF-1 clonal variants used in the present study are summarized in Table I.

**Table I** Description of RIF-1 clones

| *Clone* | *Ploidy*[1] | *Metastatic potential*[1,2] | *Doubling time*[3] *(h)* |
|---|---|---|---|
| 2 | Diploid | 11.0 | 0.84; — |
| 16 | Tetraploid | 1.1 | 0.82; 0.74 |
| 19 | Tetraploid | 67.4 | 0.74; 0.61 |
| 20 | Tetraploid | 29.1 | 0.78; 0.87 |
| 23 | Diploid | 12.8 | 0.59; 0.66 |
| 21 | Octoploid | 9.8 | 1.34; — |
| 28 | Diploid | 0.35 | 0.72; 0.94 |

[1]Reeve & Twentyman, 1983 (previous paper).
[2]Mean number lung colonies per set of lungs following i.v. injection of $10^5$ cells. Values represent the mean of 2 independent experiments. For individual determinations and s.e. values see Reeve & Twentyman, 1983.
[3]Calculated from the slope of the growth curve for cells in the exponential phase of growth.

### In vitro *radiation sensitivity*

RIF-1 clonal variants of different ploidy levels and metastatic abilities were exposed to single doses of x-radiation ranging from 1.5–16.5 Gy during log phase monolayer culture in 25 $cm^2$ tissue culture flasks (Sterilin) containing Eagles Minimal Essential Medium with Earle's salts supplemented with 10% newborn calf serum (both Gibco Biocult Ltd.) and antibiotics. Immediately after irradiation, the cells were removed from the tissue culture flasks using trypsin as previously described (Twentyman *et al.*, 1980), counted and various cell numbers were plated into replicate petri dishes containing medium. Colonies were incubated for 13 days, then fixed and stained with crystal violet. Colonies containing $\geqslant$ 50 cells were counted by means of a dissecting microscope.

A computer program (Watson, 1978) was used to derive the survival parameters Do and n according to a linear transform of the multitarget, single hit radiation survival curve.

### In vitro *drug sensitivity*

RIF-1 clones, growing in log phase monolayer culture in 25 $cm^2$ tissue culture flasks, were exposed to appropriate concentrations of the drug under study for 1 h. Adriamycin (Pharmitalia Ltd., Italy) was dissolved in sterile PBS, CCNU (Lundbeck Ltd., Luton) in absolute ethanol and melphalan (Burroughs Wellcome Co., London) in acid ethanol. All drugs were prepared immediately before use and added to the 5 ml of medium overlying the cells in a volume of 0.1 ml. As a control for each drug under study 0.1 ml of the appropriate vehicle alone was added to similar cultures. Immediately after treatment the cells were rinsed twice, trypsinized, counted and plated into replicate petri dishes as described above. Colonies were incubated for 13 days and counted.

### In vivo *drug sensitivity*

Tumours were established from RIF-1 clones by inoculating $10^5$ cells into the gastrocnemius muscle of the hind leg. Tumours were treated when they had reached ~ volume of 300–600 $mm^3$. Each mouse was ear tagged, assigned to treatment groups on a random basis and treated individually, Immediately before use CCNU was dissolved in absolute ethanol, diluted 1:20 in 0.5% carboxymethyl cellulose/Hanks Balanced Salt Solution (HBSS). Prior to injection melphalan was dissolved in acidified ethanol, diluted 1:10 in propylene glycol-$K_2HPO_4$ buffer. Appropriate concentrations of either CCNU or melphalan were injected i.p. Control mice received appropriate volumes of vehicle alone.

### *Clonogenic cell survival*

Tumour bearing mice were killed 24 h after treatment. Two tumours from mice receiving the same drug treatment were aseptically excised, pooled, weighed and disaggregated to yield single cell suspensions as previously described (Reeve & Twentyman, 1982). Various cell numbers of each suspension were plated into triplicate petri dishes and the cell colonies were counted 13 days later. Cell killing *in vivo* was expressed as surviving fraction (SF).

$$SF = \frac{\text{number of colonies formed by 100 cells from treated tumour}}{\text{number of colonies formed by 100 cells from untreated tumour}}$$

### Results

### *X-ray responses of parent RIF-1 cells and clonal sublines*

Figure 1 shows that the x-ray survival curves typically obtained for the parent RIF-1 tumour and the clonal sublines of differing ploidy levels are

**Figure 1** Typical X-ray survival curves obtained for RIF-1 parent cell line and clones of different ploidy values and metastatic capacities. In the examples shown: (◆) RIF-1 parent; (●) clone 23 (diploid; intermediate LCFE); △ clone 19 (tetraploid; high LCFE); (■) clone 21 (octoploid; low LCFE). Each point represents the mean survival value obtained from 3 replicate cultures in a single experiment.

**Figure 2** Radiosensitivities (Do) calculated from a linear transform of the multitarget X-ray survival curve of RIF-1 parent cell line and RIF-1 clones of different ploidy values and metastatic capacities. Each point represents the Do value obtained from a single experiment; error bars represent upper and lower 95% confidence limits. (◆) RIF-1 parent; (●) clone 23 (diploid; intermediate LCFE); (▲) clone 28 (diploid; low LCFE); (○) clone 16 (tetraploid; low LCFE); (△) clone 19 (tetraploid; high LCFE); (□) clone 20 (tetraploid; intermediate LCFE); ■ clone 21 (octoploid; low LCFE).

similar in shape. Figure 2 shows that the x-ray sensitivities (Do i.e. a measure of the slope) of both the parent cell line and the RIF-1 clones were within the range (1.0–2.0 Gy) usually observed for mammalian cells and that there is no consistent change in Do with increasing ploidy level. Similarly there is no consistent difference in the n values (i.e. the zero dose intercept) obtained for RIF-1 clones of different ploidy levels and the parent line (Figure 3).

### In vitro *sensitivity of RIF-1 clones to cytotoxic drugs*

*Adriamycin* Over the dose range studied (0–20$\mu$g ml$^{-1}$), tumour cell survival *in vitro* in the presence of adriamycin was similar for each of the clonal lines and was independent of clonal variation in ploidy level and metastatic ability.

*Melphalan* The data showing the response of RIF-1 clones to melphalan treatment *in vitro* are shown in Figure 4. A wide range of sensitivities to this drug over the dose range studied is evident. Thus clone 20 is considerably more resistant to melphalan than clone 16 which is most sensitive to this drug. Clones 28 and 19 show intermediate sensitivities. The observed differences in sensitivities of the clonal lines are again independent of ploidy level and metastatic ability (Table II).

*CCNU* Figure 5 shows that over the dose range studied cell survival *in vitro* in the presence of CCNU was similar for the tetraploid clones 16, 19 and 20. However, the diploid clone, 28, is significantly more sensitive to this agent. No correlation exists between the metastatic abilities of

**Figure 3** Extrapolation numbers ($n$) calculated from a linear transform of the multitarget X-ray survival curve obtained for RIF-1 parent cell line and RIF-1 clones of different ploidy values and metastatic capacities. Each point represents the $n$ value obtained from a single experiment; error bars represent upper and lower 95% confidence limits. (◆) RIF-1 parent; (●) clone 23 (diploid; intermediate LCFE); (▲) clone 28 (diploid; low LCFE); (○) clone 16 (tetraploid; low LCFE); (△) clone 19 (tetraploid; high LCFE); (□) clone 20 (tetraploid; intermediate LCFE); (■) clone 21 (octoploid; low LCFE).

**Figure 4** Cell survival curves of RIF-1 clones of different ploidy values and metastatic capacities following *in vitro* treatment with melphalan. Each point represents the survival value obtained from a single experiment. (■) clone 20 (tetraploid; intermediate LCFE); (○) clone 19 (tetraploid; high LCFE); (●) clone 28 (diploid; low LCFE); (▲) clone 16 (tetraploid; low LCFE).

the clones and their *in vitro* response to CCNU (Table II).

The data shown in Figure 6 and Table II show that the *in vitro* sensitivity to CCNU treatment exhibited by the diploid clone 28 similarly characterizes diploid clones 2 and 23. Clone 19 was used as a standard throughout these experiments (Figure 5) to typify the relative resistance of tetraploid clones to CCNU treatment.

**Table II** Metastatic potential, ploidy and cytotoxic drug sensitivity of RIF-1 clones

| *Clone* | *Metastatic Potential*[1] | *Ploidy* | *Relative*[2] *CCNU sensitivity* | *Relative*[3] *melphalan sensitivity* |
|---|---|---|---|---|
| 16 | Low | Tetraploid | Resistant | Sensitive |
| 19 | High | Tetraploid | Resistant | Intermediate |
| 20 | Intermediate | Tetraploid | Resistant | Resistant |
| 28 | Low | Diploid | Sensitive | Intermediate |
| 23 | Intermediate | Diploid | Sensitive | — |
| 2 | Intermediate | Diploid | Sensitive | — |

[1]For definition see **Table I**.
[2]See **Figure 5**.
[3]See **Figure 4**.

Figure 7 shows that over the dose range studied, cell survival *in vitro* in the presence of CCNU was similar for tetraploid clone 20 and octoploid clone 21. Both are markedly more resistant to CCNU treatment than diploid clone 28. However, octoploid clone 21 is no more resistant to *in vitro* treatment with CCNU than tetraploid clone 20.

### In vivo *sensitivity of RIF-1 clones to chemotherapeutic agents*

We have examined whether the observed clonal variation in response to melphalan and CCNU treatment *in vitro* was also detectable *in vivo*.

**Figure 5** As for **Figure 4** except following *in vitro* treatment with CCNU.

**Figure 6** Cell survival curves of tetraploid clone 19 (○), diploid clone 2 (▽) and diploid clone 23 (▼) following *in vitro* treatment with CCNU. Each point represents the survival value obtained from a single experiment.

(a) *Melphalan* Figure 8 shows data for the *in vitro* cell survival response of RIF-1 clones treated *in vivo* with melphalan. Within the range of doses studied clone 20 was less sensitive than clones 16, 19 and 28 with clone 16 being the most sensitive to melphalan.

(b) *CCNU* Figure 9 shows data for the *in vitro* cell survival response of RIF-1 clones treated *in vivo* with CCNU. Within the range of doses studied diploid clone 28 was most sensitive to CCNU treatment with tetraploid clones 16, 19 and 20 being considerably less sensitive.

### Discussion

We have examined the effect of ploidy and metastatic ability on the responses of RIF-1 clonal subpopulations to x-radiation and cytotoxic agents.

A number of clinical observations (De, 1961; Atkin *et al.*, 1959) as well as some *in vivo* and *in vitro* radiobiological studies (Revesz & Norman, 1960; Puck, 1960; Berry, 1963) have suggested a relationship between ploidy and radiation response. However, in the present study no relationship between ploidy and Do values was observed for ploidy variants of the RIF-1 tumour. Similarly no relationship was found between the extrapolation number and ploidy of cells having diploid, tetraploid or octoploid values. Our findings are in agreement with other *in vitro* studies (Till, 1961; Lockart *et al.*, 1961; Rommelaire & Errera, 1972; Limbosh *et al.*, 1974; Millar & Millar, 1977) which indicate that chromosome number *per se* does not correlate with radiation response.

For drug response, no significant differences in the dose response curves of RIF-1 clones treated with adriamycin was observed. However, our data demonstrate wide variation in the responses of RIF-1 clones to *in vitro* melphalan and CCNU treatment. Furthermore, the relative sensitivities *in vitro* are correlated with *in vivo* sensitivities in terms of clonogenic cell survival assayed *in vitro*. This latter finding contrasts with lack of correlation between *in vitro* and *in vivo* drug sensitivities observed for tumour cell subpopulations derived from a mammary adenocarcinoma (Heppner *et al.*, 1978).

The clonal variation in drug sensitivity observed for melphalan is independent of ploidy. Thus, the

**Figure 7** Cell survival curves of octoploid clone 21 (□); tetraploid clone 20 (■) and diploid clone 28 (●). Each point represents the survival value obtained from a single experiment. Poisson errors on the individual points are small compared to the inter-experimental variation. —— line drawn through data for clone 20 in **Figure 5**. ---- line drawn through data for clone 28 in **Figure 5**.

**Figure 8** Clonogenic cell survival following *in vivo* treatment of RIF-1 clones grown as solid tumours in C3H/Km mice and treated with melphalan. Poisson errors on the individual points are small compared to the inter-experimental variation. (■) clone 20 (tetraploid; intermediate LCFE); (○) clone 19 (tetraploid; high LCFE); (●) clone 28 (diploid; low LCFE); (▲) clone 16 (tetraploid; low LCFE); Each point represents the survival value obtained from a single experiment.

resistance of tetraploid clone 20 to this drug, is not shared by tetraploid clones 16 and 19. Clone 16 shows greatest sensitivity to melphalan being more sensitive than diploid clone 28 and tetraploid clone 19. A good correlation, however, exists between CCNU sensitivity and ploidy. Thus, all 3 diploid RIF-1 clones were significantly more sensitive to *in vitro* CCNU treatment than tetraploid or octoploid clones.

Whilst a number of studies have demonstrated the presence of subpopulations of tumour cells with markedly different drug sensitivities (Hakansson & Trope, 1974*a,b*; Palyi *et al.*, 1977; Heppner *et al.*, 1978; Tsuruo & Fidler, 1981) no-one to our knowledge, has reported ploidy-dependent drug responses. No correlation, however, between drug response and metastatic ability was observed for the RIF-1 clones examined in the present study.

Our data show that there is no correlation between the growth kinetics of the clones and their drug sensitivities. This finding is in agreement with similar observations (Hakansson & Trope, 1973; Van Putten, 1974) which also suggest that differences in clone sensitivity to drug treatment are not likely to be explained by small differences in cell proliferation. We are currently examining CCNU and melphalan transport in cells of RIF-1 subpopulations which differ in their sensitivities to these agents, together with DNA repair properties, in an attempt to elucidate the cellular differences responsible for the diverse responses of RIF-1 clones to these agents.

**Figure 9** As for **Figure 8** except following *in vivo* treatment with CCNU.

### References

ATKIN, N.M., RICHARDS, B.M. & ROSS, A.J. (1959). The deoxyribonucleic acid content of carcinoma of the uterus: An assessment of its possible significance in relation to histopathology and clinical course based on 165 cases. *Br. J, Cancer*, **13**, 773.

BERRY, R.J. (1963). Quantitative studies of relationships between tumour cell ploidy and dose response to ionizing radiation *in vivo*. *Radiat. Res.*, **18**, 236.

DE, N. (1961). Polyploidy and radiosensitive behaviour of human malignant cells *in vivo*. *Br. J. Cancer*, **15**, 54.

HAKANSSON, L. & TROPE, C. (1973). An *in vitro* study of the effect of cytostatic drugs on the DNA synthesis in methylcholanthrene-induced mouse sarcomas and in rat Walker 256 tumours. *Acta Pathol. Microbiol. Scand.*, **81**, 552.

HAKANSSON, L. & TROPE, C. (1974*a*). On the presence within tumours of clones that differ in sensitivity to cytostatic drugs. *Acta Pathol. Microbiol. Scand.*, **82**, 35.

HAKANSSON, L. & TROPE, C. (1974*b*). Cell clones with different sensitivity to cytostatic drugs in methylcholanthrene-induced mouse sarcomas. *Acta Pathol. Microbiol. Scand.*, **82**, 41.

HEPPNER, G.H., DEXTER, D.L., DENUCCI, T., MILLER, F.R. & CALABRESI, P. (1978). Heterogeneity in drug sensitivity among tumour cell subpopulations of a single mammary tumour. *Cancer Res.*, **38**, 3758.

LIMBOSH, S., HEILPORN, V., LIEVENS, A., DECOEN, J.L. & ZAMPETTI-BOSSELER, F. (1974). Radiation response of a somatic cell hybrid. *Int. J. Radiat. Biol.*, **26**, 197.

LOCKART, R.Z., ELKIN, D.M.M. & MOSES, W.B. (1961). Radiation responses of mammalian cells grown in culture. II. Survival and recovery characteristics of several subcultures of HeLa S3 cells after X-irradiation. *J. Natl Cancer Inst.*, **271**, 1393.

MILLAR, B.C. & MILLAR, J.L. (1977). The effect of ploidy on the modification of the shoulder region of hypoxic cell survival curves by the biradical, Ro 03-6061. *Int. J. Radiat. Biol.*, **31**, 355.

NOWELL, P.L. (1976). The clonal evolution of tumour cell populations. *Science*, **194**, 23.

PALYI, I., OLAH, & SUGAR, J. (1977). Drug sensitivity studies on clonal cell lines isolated from heteroploid tumour cell populations. I. Dose response of clones growing in monolayer cultures. *Int. J. Cancer*, **19**, 859.

POSTE, G. & FIDLER, I.J. (1980). The pathogenesis of cancer metastasis. *Nature*, **283**, 139.

PUCK, T.T. (1960). The action of radiation of mammalian cells. *Am. Naturalist.*, **94**, 95.

REEVE, J.G. & TWENTYMAN, P.R. (1983*a*). Clonal variation in the arrest, survival and growth of RIF-1 mouse sarcoma cells in the lungs of C3H mice. *Br. J. Cancer*, **47**.

REEVE, J.G. & TWENTYMAN, P.R. (1982). Ploidy distribution of tumour cells derived from induced and spontaneously arising metastases of a unique murine radiation-induced sarcoma, RIF-1. *Eur. J. Cancer Clin. Oncol.*, **18**, 1001.

REVESZ, L. & NORMAN, U. (1960). Chromosome ploidy and radiosensitivity of tumours. *Nature*, **187**, 361.

ROMMELAIRE, J. & ERRERA, M. (1972). The effect of caffeine on the survival of U.V. irradiated diploid and tetraploid Chinese-hamster cells. *Int. J. Radiat. Biol.*, **22**, 285.

SPOONER, M.E. & COOPER, E.H. (1972). Chromosome constitution of transitional cell carcinomas of the urinary bladder. *Cancer*, **29**, 1401.

STICH, H.F. (1960). The DNA content of tumour cells. II. Alterations during the formation of hepatomas in rats. *J. Natl Cancer Inst.*, **24**, 1283.

SUZUKI, N., WILLIAMS, M., HUNTER, N.M. & WITHERS, H.R. (1980). Malignant properties and DNA content of daughter clones from a mouse fibrosarcoma: differentiation between malignant properties. *Br. J. Cancer*, **42**, 765.

SUZUKI, N., WITHERS, H.R. & LEE, L.Y. (1977). Variability of DNA content of murine fibrosarcoma cells. *Nature*, **269**, 251.

SUZUKI, N., WITHERS, H.R. & WILLIAMS, N. (1978). Heterogeneity and variability of artificial lung colony forming ability among clones from mouse fibrosarcoma. *Br. J. Cancer*, **38**, 3349.

TILL, J.E. (1961). Radiosensitivity and chromosome number in strain L mouse cells in tissue culture. *Radiat Res.*, **15**, 400.

TSURUO, T. & FIDLER, I.J. (1981). Differences in drug sensitivity among tumour cells from parental tumours, selected variants, and spontaneous metastases. *Cancer Res.*, **41**, 3058.

TWENTYMAN, P.R., BROWN, J.M., GRAY, J.W., FRANKO, A.J., SCOLES, M.A. & KALLMAN, R.F. (1980). A new mouse tumour model system (RIF-1) for comparison of endpoint studies. *J. Natl Cancer Inst.*, **64**, 595.

VAN PUTTEN, L.M. (1974). Are cell kinetic data relevant for the design of tumour chemotherapy schedules? *Cell Tissue Kinet.*, **7**, 493.

WATSON, J.V. (1978). A linear transform of the multi-target survival curves. *Br. J. Radiol.*, **51**, 534.